# Can fitness testing promote physical activity in adolescents with juvenile idiopathic arthritis?

**DOI:** 10.1186/s12969-026-01223-6

**Published:** 2026-05-21

**Authors:** Ulrika Nilsson, Marianne Skattør, Helga Sanner, Kristine Risum

**Affiliations:** 1 Section for Orthopaedic Rehabilitation Rikshospitalet, Division of Orthopaedic Surgery, Oslo University Hospital, Oslo, Norway; 2 Department of Rheumatology, Oslo University Hospital Rikshospitalet, Oslo, Norway; 3 Institute of Clinical Medicine, Medical Faculty, University of Oslo, Oslo, Norway; 4 Department of Rehabilitation Science and Health Technology, Oslo Metropolitan University, Oslo, Norway

**Keywords:** Juvenile idiopathic arthritis, Physical activity, Cardiorespiratory fitness, Exercise test

## Abstract

**Background:**

Physical activity (PA) is important for disease management and overall health in adolescents with juvenile idiopathic arthritis (JIA). In clinical practice, identifying low cardiorespiratory fitness (CRF) and encouraging higher-intensity PA are priorities. We investigated whether introducing submaximal treadmill fitness testing could increase PA, estimated peak oxygen uptake (VO_2peak_), walking distance, physical function, and health-related quality of life (HRQoL) in adolescents with JIA, and examined associations between PA, VO_2peak_, and walking distance.

**Methods:**

In this exploratory pre–post study, adolescents aged 10–18 years with non-systemic JIA were consecutively recruited during routine physiotherapy visits at a rheumatology clinic. A submaximal treadmill test was used to estimate VO_2peak_ at baseline and follow-up. Self-reported PA was assessed with the International Physical Activity Questionnaire Short-Form (IPAQ-SF), and physical function and HRQoL with the Juvenile Arthritis Multidimensional Assessment Report (JAMAR). Within-participant changes were analysed with paired tests; associations were assessed using Spearman’s rank correlation.

**Results:**

Thirty-four participants were included; 24 completed follow-up. IPAQ-derived PA did not change significantly between assessments. Estimated VO_2peak_ was higher at follow-up by 2.4 (SD 4.4) mL∙kg^− 1^∙min^− 1^ (*P* = 0.01) and walking distance was higher by 31 (69) metres (*P* = 0.04), whereas no significant changes were observed in JAMAR physical function or HRQoL. Vigorous PA showed the strongest associations with estimated VO_2peak_ at baseline and follow-up (rho up to 0.51; *P* < 0.05). Associations of moderate-to-vigorous and total PA with VO_2peak_ were weaker and evident only at follow-up (rho ~ 0.41–0.43), and vigorous PA correlated with walking distance at follow-up (rho = 0.42; *P* = 0.040); other associations were low or non-significant.

**Conclusions:**

Introducing submaximal treadmill fitness testing in routine care did not change self-reported PA, but estimated VO_2peak_ and walking distance increased significantly. Submaximal testing may be a feasible clinical tool to identify low CRF and support counselling. Larger studies are needed to determine whether fitness testing can promote PA and to identify which adolescents benefit most, using measurement approaches that capture complementary aspects of PA (e.g., self-report for context and perceived behaviour, and device-based metrics for volume and intensity where appropriate).

**Supplementary Information:**

The online version contains supplementary material available at 10.1186/s12969-026-01223-6.

## Background

Juvenile idiopathic arthritis (JIA) is a chronic autoimmune disease of unknown aetiology with onset before the age of 16. The disease causes joint inflammation, leading to symptoms such as joint swelling, pain, and stiffness, which can affect physical function and the ability to participate in physical activity (PA) [1]. The medical treatment for JIA is anti-inflammatory medicines, with biologic agents used when indicated. The latter were introduced in 2000, providing targeted and effective treatment for patients with JIA [1]. In addition, PA may theoretically have an anti-inflammatory effect by inhibiting pro-inflammatory pathways and increasing anti-inflammatory cytokines and cortisol [2, 3]. The World Health Organization (WHO) recommends children to be physically active at moderate-to-vigorous intensity for 60 min per day [4]. Research has shown that PA does not increase disease activity in patients with JIA [5] and it is particularly important to be physically active to mitigate the consequences of the disease and reduce the risk of comorbidities. PA is considered one of seven protective cardiovascular health factors, and increased PA may decrease chronic inflammation and endothelial dysfunction [6]. Physical inactivity in this patient group may be a risk factor for early development of subclinical signs of arteriosclerosis [7].

Contrary to findings in international studies [8], Norwegian patients with JIA have shown accelerometer-measured PA levels similar to those as healthy controls [9], and similar cardiorespiratory fitness (CRF) to the control group [10]. However, in the Norwegian sample the patients spent less time in vigorous-intensity activities compared with controls [9] and 20% of girls and 33% of boys in the JIA patient group had low CRF [10]. In clinical practice, it is important to identify these patients and encourage them to engage in PA, especially vigorous-intensity activities that have the greatest impact on increasing CRF [10]. In our clinical practice, we have emphasized patient education to encourage our patients to be physically active, but fitness testing has not been routinely performed during physiotherapy assessments. Direct measurement of VO_2peak_ is considered the gold standard assessment of CRF, but it is time-consuming and requires equipment not readily available in routine clinical practice. Submaximal tests can be used to estimate VO_2peak_ and are often feasible in the clinical setting [11]. Our hypothesis was that fitness testing could serve as a tool to increase patients’ knowledge and awareness of fitness and motivate them to increase their PA levels. Therefore, we aimed to investigate whether the introduction of fitness testing, using a submaximal treadmill test, would lead to self-reported changes in PA levels (primary outcomes) among adolescents with JIA. Additionally, we sought to assess changes in estimated peak oxygen uptake (VO_2peak_), walking distance, physical function, and health-related quality of life (HRQoL) (secondary outcomes). We also aimed to investigate the association between PA and CRF in adolescents with JIA.

## Methods

### Patients

In this clinical follow-up study with a pre- and post-test design, we included adolescents with JIA aged 10–18 years. The participants were enrolled consecutively from June 2019 to September 2021. This was an exploratory clinical study; accordingly, no formal sample size calculation was undertaken due to limited paediatric data to inform expected changes and variance. Inclusion criteria were classification according to the International League of Associations for Rheumatology (ILAR) criteria for JIA [12], and the ability to complete the submaximal treadmill test and the questionnaires in Norwegian. Exclusion criteria were systemic-onset JIA, comorbidities or other conditions that made it difficult to perform the submaximal treadmill test and/or answer the questionnaires (e.g., severe heart disease, fractures, recent surgery, or language barriers).

The project was approved by the Regional Ethics Committee (REK, 2019/153) and by the Data Protection Officer at Oslo University Hospital (OUS, 19/01610). Adolescents aged 16–18 years provided their own written consent to participate in the study. Guardians of participants < 16 years provided written consent for their child to participate.

### Procedures for data collection

Eligible adolescents were identified when scheduled for routine physiotherapy as part of rheumatology follow-up at OUS and were invited consecutively, irrespective of the reason for referral. Referral to physiotherapy was based on clinical judgement (treating physician or physiotherapist scheduling follow-up at a prior visit) and was not contingent on concerns about low physical activity.

Physiotherapists affiliated with the unit conducted the submaximal treadmill tests and clinical assessments during the routine physiotherapy visits; additionally, patients completed questionnaires at baseline (Visit 1). Participants were retested at the subsequent follow-up assessment using the same treadmill test and questionnaires (Visit 2). Follow-up assessments were planned after approximately 3 months, but due to COVID-19 some took place later than planned (up to 11 months). As part of usual care, all adolescents and their families receive education about PA at diagnosis, and PA is assessed and discussed at follow-up visits, particularly when low PA is identified. In this project, we added a submaximal treadmill fitness test and provided routine PA counselling; no study-specific, structured PA counselling was delivered.

The COVID-19 pandemic affected study implementation, resulting in a prolonged inclusion period, reduced in-person attendance, and fewer referrals to physiotherapy. Several follow-up visits were postponed, and many were conducted via video consultation. Consequently, several participants did not undergo treadmill testing at Visit 2.

### Measurement methods

#### Physical activity level

Self‑reported PA was assessed using the Norwegian short‑form International Physical Activity Questionnaire (IPAQ‑SF), referring to the last 7 days [13].

Participants reported days per week and minutes per day spent walking, in moderate‑intensity and in vigorous‑intensity activity; minutes per day were derived for each category. Moderate‑to‑vigorous PA (MVPA) was calculated as the sum of moderate and vigorous minutes. As a sensitivity analysis, we calculated metabolic equivalent of task (MET)‑minutes per day for walking (3.3 METs), moderate (4.0 METs), vigorous (8.0 METs), MVPA, and total PA; MET‑minutes per day were computed as MET value × minutes per day following the IPAQ protocol [14]. IPAQ-SF was our primary outcome measure.

There are no studies of measurement properties of the IPAQ‑SF in adolescents with JIA. In studies in other paediatric rheumatic diaseses (juvenile dermatomyositis, JDM and juvenile systemic lupus erythematosus, JSLE) and in healthy children and adolescents, the IPAQ‑SF has shown poor agreement with criterion measures and moderate test–retest reliability in certain subgroups [15–17]. In adults, the IPAQ-SF has shown acceptable reliability and criterion validity for vigorous activity and sitting; moderate reliability for walking and fair reliability for moderate activity; and moderate correlations with VO_2peak_ [18].

#### Submaximal treadmill test

We used the submaximal treadmill test developed by Ebbeling et al. [19] to estimate VO_2peak_, using a treadmill (Technogym, Rimini, Italy). During the first four minutes, the adolescents walked at 0% incline at a speed between 3.2 km/h (2.0 mph) and 7.2 km/h (4.5 mph) to elicit a heart rate (HR) between 50% and 70% of age‑predicted peak HR (HR_peak_) calculated as 220 − age. If possible, we aimed for a HR close to 70% of predicted HR_peak_, increasing the speed gradually until this intensity was reached. If an HR close to 70% of predicted HR_peak_ was not reached by 7.2 km/h (4.5 mph), the highest attainable speed was used, and the corresponding HR was recorded. After four minutes, the treadmill incline was then increased over 15–20 s to 5% for the next four minutes. HR was measured at the end of each stage using a HR monitor (Polar, Kempele, Finland). Participants rated their perceived exertion (RPE) using the Borg 6–20 scale [20] at 3 and 8 min. Participants who were unfamiliar with treadmill walking received a brief familiarisation prior to testing to ensure comfort.

VO_2peak_ was estimated using a predetermined formula based on walking speed at eight minutes, age, sex, and HR at eight minutes [19]. We also recorded the total walking distance (m) the adolescents walked during the submaximal treadmill test.

The test has been validated and tested for reliability in adult patients with rheumatic diseases [21]. In patients with JIA, a study demonstrated that the test is suitable for research, showing group-level validity [22]. The test can be used to assess changes in fitness, when the same relative HR percentage is applied pre- and post-training [23].

#### Juvenile Arthritis Multidimensional Assessment Report (JAMAR)

JAMAR is a patient-reported questionnaire that assesses multiple aspects of living with JIA. In this study, we used the physical function domain (Juvenile Arthritis Functionality Scale, JAFS; 15 items, scored from 0 = without difficulty to 3 = unable to do), and HRQoL domain (The Pediatric Rheumatology Quality of Life Scale, PRQL; 10 items, scored from 0 = never to 3 = all the time), each summarized as total scores (0–45, 0 = best function, and 0–30, 0 = best HRQoL, respectively) [24]. The adolescents completed the questionnaire independently, with assistance from their guardians if needed.

Joint status (swelling, range of motion, and pain) was assessed as a routine part of the physiotherapy assessment. The Physician Global Assessment of disease activity (PGA) was completed by the treating physician on a 0–100 mm visual analogue scale, with higher scores indicating higher disease activity [25, 26]. Background variables, including demographic characteristics, diagnosis, comorbidities, disease activity, medication use, and participation in physical education (PE) in school and leisure-time PA, were obtained from patients and medical records.

### Statistical analyses

Demographic data are presented as mean (SD), median (25th-75th percentile), or n (%) as appropriate. Paired tests (t-tests or Wilcoxon signed-rank tests, as appropriate) were used to examine changes in PA levels, estimated VO_2peak_, walking distance, physical function, and HRQoL, as well as other outcome measures for patients who completed the retest. Unpaired tests were used to compare patients who completed the retest (*n* = 24) with those who had baseline only (*n* = 10) to assess representativeness. Associations between PA, estimated VO_2peak_, and walking distance were examined using Spearman’s rank correlation (rho). We interpreted correlations as weak (< 0.30), moderate (0.30 to 0.60), and high (> 0.60) [27]. Significance levels were set to *P* < 0.05 in all analyses. Given the exploratory nature, P-values were not adjusted for multiple comparisons and findings were interpreted cautiously. SPSS version 30 was used for statistical analyses (IBM Corp., Armonk, NY, USA).

## Results

### Patient characteristics

A total of 34 adolescents with JIA were included in the study, of whom 24 patients completed the retest at Visit 2 (Table 1). Among those who completed the study (*n* = 24), the mean (SD) age was 14.4 (2.2) years, and 15 (63%) were girls. In this group, 12 (50%) patients participated in organised PA, and 19 (79%) participated fully in physical education. Among participants assessed only at Visit 1 (*n* = 10), the mean (SD) age was 13.8 (2.6) years, and 6 (60%) were girls; eight (80%) patients participated in organised PA, and five (50%) fully participated in PE. The disease activity assessed by PGA was low and comparable in both groups (Table 1).

**Table 1:** Demographic characteristics

		Patients with complete dataset (*n* = 24)	Patients with data at visit 1 only (*n* = 10)
Age		14.4 (2.2)	13.8 (2.6)
Sex (m/f), n (%)		9/15 (38/63)	4/6 (40/60)
ILAR classification, n (%)			
Persistent oligo JIA		8 (33)	5 (50)
Extended oligo JIA		1 (4)	1 (10)
Poly JIA RF-		7 (29)	1 (10)
ERA JIA		6 (25)	2 (20)
PsA		2 (8)	1 (10)
Height		164.6 (12.5)	162.8 (10.7)
Weight		60.2 (15.7)	57.0 (12.5)
BMI		22.0 (4.3)	21.3 (3.3)
Disease duration		2.9 (3.8)	3.2 (3.0)
PGA (0–100, 0 = best)		10.0 (13.9)	17.2 (21.9)
Participation organized PA		12 (50)	8 (80)
Participation unorganized PA		16 (67)	6 (60)
Participation in PE			
Always		19 (79)	5 (50)
Always (with some modifications)		3 (13)	4 (40)
Sometimes (with some modifications)		1 (4)	0 (0)
Seldom/never		1 (4)	1 (10)
Local physiotherapy		4 (17)	3 (30)
IPAQ-SF			
Walking, minutes per day		18 (13, 56)	25 (2, 46)
Moderate PA, minutes per day		17 (4, 34)	17 (10, 26)
Vigorous PA, minutes per day		28 (7, 62)	21 (12, 32)
MVPA, minutes per day		56 (26, 98)	41 (30,50)
Total PA, minutes per day		83 (41, 144)	60 (46, 88)
Sitting, minutes per weekday		480 (390, 600)	570 (345, 608)
Submaximal CRF			
Estimated VO_2peak_ (mL∙kg^− 1^∙min^− 1^)		42.2 (13.0)	43.4 (10.6)
Walking distance (m)		733 (136)	691 (86)
Speed (km/h)		5.9 (1.0)	6.0 (0.7)
3 min	HR (beats/min)	131 (13)	126 (14)
	Borg _6−20_	10.6 (2.0)	11.0 (2.1)
8 min	HR (beats/min)	162 (20)	162 (17)
	Borg _6−20_	13.2 (2.1)	13.6 (2.2)
1 min post test	HR (beats/min)	126 (23)	127 (16)
2 min post test	HR (beats/min)	115 (20)	115 (13)
PRQL total score (0–30, 0 = best)		4.00 (1.25, 8.75)	4.50 (2.00, 8.00)
JAFS total score (0–45, 0 = best)		1.00 (0.00, 5.00)	3.00 (0.75, 5.75)

Numbers are mean (SD) or median (25th -75th percentiles) unless otherwise stated. *BMI* body mass index, *CRF* cardiorespiratory fitness, *ERA* enthesitis-related arthritis, *JAFS* juvenile arthritis functional scale, *JAMAR* juvenile arthritis multidimensional assessment report, *HRQoL* health-related quality of life, *HR* heart rate, *ILAR* international league against rheumatism, *IPA*Q*-SF* The International Physical Activity Questionnaire Short-Form, *JIA* juvenile idiopathic arthritis, *MVPA* moderate-to-vigorous physical activity, *PA* physical activity, *PE* physical education, *PGA* physician’s global assessment, *PR*Q*L* The Pediatric Rheumatology Quality of Life Scale, *PsA* psoriatic arthritis, *VO*_*2peak*_ peak oxygen uptake

### Changes in PA levels, VO_2peak_, walking distance, physical function and HRQoL between Visit 1 and Visit 2

There were no statistically significant changes in IPAQ-SF measured PA variables between Visit 1 and Visit 2 (Table 2). These included daily minutes of walking, moderate, vigorous, MVPA, and total PA. However, non-significant trends towards improvement were observed and should be interpreted with caution. There was a statistically significant change in estimated VO_2peak_ (mean (SD) 2.4 (4.4) mL∙kg-1∙min-1, *P* = 0.012) and walking distance (mean (SD) 31 (69) metres, *P* = 0.038) between visits (Table 3). There were no statistically significant changes in HRQoL or physical function measured with JAMAR. However, for all variables without statistically significant changes, clear trends toward improvement were observed, suggesting insufficient statistical power.

**Table 2:** Changes in PA levels

	Visit 1 (*n* = 24)	Visit 2 (*n* = 24)	Change	*P*-value
IPAQ-SF				
Walking, minutes per day	18 (13, 56)	14 (6, 28)	-5 (-19, 15)	0.529
Moderate PA, minutes per day	17 (4, 34)	19 (3, 34)	2.5 (-21, 18)	0.543
Vigorous PA, minutes per day	28 (7, 62)	39 (13, 61)	1 (9, 21)	0.498
MVPA, minutes per day	56 (26, 98)	73 (17, 90)	0 (-27, 49)	0.658
Total PA, minutes per day	83 (41, 144)	96 (31, 129)	-2 (-38, 59)	0.909
Sitting, minutes per day	480 (390, 600)	480 (308, 540)	0 (-180, 240)	0.403

Numbers are median (25th -75th percentiles). *IPAQ-SF* The International Physical Activity Questionnaire Short-Form, *MVPA* moderate-to-vigorous physical activity, *PA* physical activity

**Table 3:** Changes in estimated VO_2peak_, physical function and HRQoL

		Visit 1 (*n* = 24)	Visit 2 (*n* = 24)	Change	*P*-value
Submaximal CRF					
Estimated VO_2peak_ (mL∙kg^− 1^∙min^− 1^)		42.2 (13.0)	44.7 (12.9)	2.4 (4.4)	**0.012**
Walking distance (m)		733 (136)	764 (120)	31 (69)	**0.038**
Speed (km/h)		5.9 (1.0)	6.1 (0.9)	0.2 (0.6)	0.110
3 min	HR (beats/min)	131 (13)	129 (13)	2 (15)	0.297
	Borg _6−20_	10.6 (2.0)	10.9 (1.6)	0.3 (2.5)	0679
8 min	HR (beats/min)	162 (20)	163 (17)	1 (21)	0.520
	Borg _6−20_	13.2 (2.1)	13.8 (1.4)	0.5 (2.4)	0.318
1 min post test	HR (beats/min)	126 (23)	124 (18)	2 (19)	0.284
2 min post test	HR (beats/min)	115 (20)	112 (16)	3 (18)	0.171
JAMAR					
PRQL total score (0–10)		4.00 (1.25–8.75)	3.00 (1.00–7.00)	0.00 (-2.00, 0.00).	0.370
JAFS total score		1.00 (0.00-4.25)	0.00 (0.00–3.00)	0.00 (0.2.00, 1.00)	0.113

Numbers are mean (SD) or median (25th -75th percentiles). *CRF* cardiorespiratory fitness, *JAFS* juvenile arthritis functional scale, *JAMAR* juvenile arthritis multidimensional assessment report, *HR* heart rate, *HRQoL* health-related quality of life, *PRQL* The Pediatric Rheumatology Quality of Life Scale, *VO*_*2peak*_ peak oxygen uptake

### Associations between PA levels, VO_2peak_, and walking distance

At Visit 1 (*n* = 34), there was a moderate, positive correlation between vigorous PA and estimated VO_2peak_ and total PA and estimated VO_2peak_ (Table 4). Correlations of walking and moderate PA and estimated VO_2peak_ were weak and not statistically significant. There were weak, non-significant correlations between all PA variables and walking distance (all rhos < 0.30, all *P* > 0.05) (Table 4).

**Table 4:** Associations between PA, estimated VO_2peak_ and walking distance

	Baseline (Visit 1) (*n* = 34)		Follow-up (Visit 2) (*n* = 24)	
	Estimated VO_2peak_ (mL∙kg^− 1^∙min^− 1^)	Walking distance (m)	Estimated VO_2peak_ (mL∙kg^− 1^∙min^− 1^)	Walking distance (m)
Walking, minutes per day	0.11	0.06	0.04	0.13
Moderate PA, minutes per day	-0.10	0.14	0.08	0.08
Vigorous PA, minutes per day	0.36*	0.17	0.51*	0.42*
MVPA, minutes per day	0.26	0.23	0.43*	**0.32**
Total PA, minutes per day	0.34*	0.28	0.41*	**0.39**
Sitting, minutes per day	-0.02	0.24	-0.17	-0.19

Numbers are Spearman’s Rho, moderate correlations in bold, *p < 0.05

*MVPA* moderate-to-vigorous physical activity, *PA* physical activity; *VO*_*2peak*_ peak oxygen uptake

At Visit 2 (*n* = 24), there were significant, moderate positive correlations between vigorous PA and estimated VO_2peak_, MVPA and estimated VO_2peak_, total PA and estimated VO_2peak_, and vigorous PA and walking distance (Table 4). There were moderate but not statistically significant correlations between MVPA and walking distance and total PA and walking distance. Walking and PA showed weak, non-significant correlations with both estimated VO_2peak_ and walking distance (Tabel 4).

Among participants with data at both visits, we found weak correlations between changes in PA levels and changes in V0_2peak_ and walking distance (all rhos < 0.30, *P* > 0.05) (Table 5).

**Table 5:** Associations between changes in PA, changes in estimated VO_2peak_ and changes in walking distance between Visit 1 and Visit 2

(*n* = 24)	Estimated VO_2peak_ (mL∙kg^− 1^∙min^− 1^)		Walking distance (m)	
	Spearman’s rho	*p*-value	Spearman’s rho	*p*-value
Walking, minutes per day	0.24	0.27	0.152	0.48
Moderate PA, minutes per day	-0.07	0.74	0.041	0.85
Vigorous PA, minutes per day	0.11	0.60	-0.053	0.80
MVPA, minutes per day	0.01	0.95	-0.002	0.99
Total PA, minutes per day	0.14	0.51	0.085	0.69
Sitting, minutes per day	-0.16	0.45	0.085	0.70

*MVPA* moderate-to-vigorous physical activity, *PA* physical activity, *VO*_*2peak*_ peak oxygen uptake

Findings were consistent when PA was expressed as MET‑minutes per day: no statistically significant differences were observed between visits, and correlations between MET‑based PA and estimated VO_2peak_ and walking distance mirrored those obtained using minutes per day (Supplementary Tables S1-S4).

## Discussion

To our knowledge, this is the first study to investigate whether the introduction of fitness testing using a submaximal treadmill test could increase PA levels, CRF, physical function and HRQoL among adolescents with JIA. In this small exploratory cohort, introducing submaximal treadmill testing within routine care did not result in statistically significant changes in self-reported PA between Visit 1 and Visit 2, although there was a trend towards improvement. We found a significant increase in estimated VO_2peak_ and walking distance between Visit 1 and Visit 2, while physical function and HRQoL did not change significantly. Correlations supported a physiological link: vigorous PA showed the strongest associations with estimated VO_2peak_ and, to a lesser extent, walking distance, whereas associations for walking and moderate activity were low or non‑significant; correlations between changes in these measures were negligible.

We were unable to find an identical study for direct comparison. In a pilot study, a wearable activity tracker was used to promote PA in adolescents with JIA [28]. Their results were consistent with ours as they found no significant differences in PA levels from week 1 to week 5 after introducing the activity tracker to the adolescents [28]. A systematic review reported that wearable activity trackers could increase PA levels in children and adults with chronic musculoskeletal conditions [29]. Despite differences in study design compared to our study, all studies examined whether measuring or monitoring physical parameters could influence patients’ PA levels. We used the IPAQ-SF to measure PA because it is feasible and widely used and is comparable to other self-reported tools [30]. However, in pediatric populations IPAQ-SF shows poor agreement with criterion measures, so estimates should be interpreted with caution [15–17]. As for all self-reported measures of PA levels, it relies on participants’ understanding and recall and may therefore capture PA differently from accelerometry.

Our study was conducted during a period of pandemic-related restrictions due to COVID-19, which variably affected school attendance, PE delivery and access to organised PA across municipalities (periods of remote schooling and modified, home-based PE), potentially influencing participation and self-reported PA. We did not systematically capture municipality-level restrictions. Another study conducted during the COVID-19 pandemic reported that most children and adolescents with JIA, JDM, and JSLE were classified as physically active measured by the IPAQ-SF on the basis of moderate-intensity PA alone; none reported any vigorous PA [31]. By contrast, our participants reported median 28 min/day of vigorous PA at inclusion. In Norway, school PE prioritises participation over routine fitness testing. Tests, if used, are pedagogical rather than graded. Clinic‑based submaximal testing can therefore provide novel CRF feedback to support counselling. School is a key venue for peer‑based PA. Although most participants in our cohort reported full participation in school PE, some adolescents may still need targeted support.

There was a significant increase in estimated VO_2peak_ and walking distance between visits. Although no prior studies have tested whether introducing a submaximal fitness test can improve fitness in adolescents with JIA, related work suggests that measurement itself may prompt gains. In one study including patients with JIA, 6-minute walk distance improved before a pedometer intervention commenced, likely reflecting accountability and support [32], and in another including women with SLE, repeated assessments were associated with increased PA and aerobic capacity in both the intervention and control groups [33]. Introducing submaximal testing may thus have functioned as a brief intervention - raising awareness through immediate feedback and facilitating counselling. Evidence from paediatric clinics has indicated that fitness assessment is most effective when combined with brief counselling, individualised goal-setting and/or structured programmes, improving CRF and supporting behaviour change [34, 35]. Feasibility will vary across clinics; where treadmills are unavailable or time is limited, field tests suited to local resources, such as the 6-minute walk test can be used, noting they reflect walking capacity rather than VO_2peak_ [36].

Although our mean increase in estimated VO_2peak_ (2.4 mL·kg − 1·min − 1) is below the frequently cited 3.5 mL·kg − 1·min − 1 benchmark from adult epidemiology [37], even smaller improvements (approximately 1 mL·kg − 1·min − 1) have been associated with meaningful health benefits [38]. Thus, the observed change may be clinically meaningful. Yet, adult‑based thresholds may not translate directly to adolescents with JIA. Importantly, our mean change exceeded the group‑level minimal detectable change (MDC_95_) previously reported for this submaximal treadmill protocol (1.5 mL·kg − 1·min − 1 for test–retest; 1.1 mL·kg − 1·min − 1 for inter‑rater), supporting that the observed improvement is likely beyond measurement error in this setting [22].

Correlations were strongest for vigorous PA with estimated VO_2peak_ at both visits. There was also a positive moderate correlation between moderate-to-vigorous PA and estimated VO_2peak_ at Visit 2. This is consistent with our previous findings in adolescents with JIA and controls [10], underscoring the importance of vigorous-intensity PA for CRF.

Our patients did not report statistically significant changes in physical function (JAFS) and HRQoL (PRQL) measured with JAMAR. Baseline scores for both outcomes were good, leaving little room for improvement in our sample. Especially for JAFS, with the median total score of 1.00 at Visit 1, there was clustering at the better-function end of the scale. The ceiling effect of JAFS makes it less sensitive to change in milder cases with low levels of disability, reducing its ability to detect improvements in function for patients who are close to or at the ceiling [39].

This study has several limitations that should be acknowledged. Unfortunately, we did not record individuals who declined to participate. However, we recruited participants consecutively in our hospital clinic, reflecting the population typically referred to physiotherapy. Enrolment via physiotherapy follow-up at a single tertiary centre limits generalisability to adolescents with JIA receiving physiotherapy in similar settings and may not extend to those not referred. The study was exploratory, without an a priori sample size calculation due to insufficient paediatric data, with a modest sample (*n* = 34; 24 [70.6%] completed) and COVID-19–related attrition (postponements/telehealth precluding on-site treadmill testing); no exercise intervention was delivered. Completers and non-completers were comparable at baseline (PA, estimated VO_2peak_, walking distance, age, sex, ILAR), reducing attrition bias; nevertheless, attrition and sample size limit statistical power, particularly for detecting small changes in self-reported PA, and selection bias cannot be excluded.

In our study, the retest was planned to occur after 3–6 months. Due to the COVID‑19 pandemic, the timing of Visit 2 follow‑up assessments was variable, and some occurred later than planned. We cannot rule out that this may have influenced the results. Regarding measurement methods, we have already discussed limitations relating to the IPAQ-SF. We used a submaximal treadmill test to assess fitness. The gold standard for assessing CRF is direct measurement of VO_2peak_ to volitional exhaustion during treadmill or cycle ergometer testing [11]. However, such testing is time‑consuming, requires specialised equipment, and is more demanding for patients. In our study, we chose a submaximal treadmill test primarily because it is feasible and easy to implement in clinical practice. The submaximal treadmill test has shown acceptable criterion validity at the group level, but not at the individual level, in adolescents with JIA [22]. We did not adjust for age-related development because the VO_2peak_ prediction equation includes age. Given the follow-up interval, maturation effects are likely small but cannot be excluded. Although many participants were accustomed to treadmill walking and familiarisation was provided where needed, a practice effect at retest remains possible.

## Conclusions

In conclusion, we observed significant increases in estimated VO_2peak_ and walking distance between baseline and follow-up in this cohort of adolescents with JIA during a period when a submaximal treadmill fitness test was incorporated into routine care. Although PA levels did not increase significantly, several PA parameters showed positive trends. Future research should aim to identify effective strategies to motivate and facilitate PA among adolescents with JIA and to determine which patients might benefit most from targeted interventions. For this purpose, a submaximal treadmill test might be a valuable, feasible tool in clinical practice.

## Supplementary Information

Below is the link to the electronic supplementary material.

Supplementary Material 1

## Data Availability

The dataset generated and analyzed during the current study is not publicly available due to strict ethical regulation of health-related data in Norway. The consent to participate does not include permission to make the data available to a third party.

## Declarations

### Use of AI

We used ChatGPT (OpenAI, San Francisco, CA, USA) for grammar and spell checking; the authors are responsible for the content and interpretation.

## Abbreviationsnull

ERA: Enthesitis-releated arthrisi
JIA: Juvenile idiopathic arthritis
HR: Heart rate
HR_peak_: Peak heart rate
HRQoL: Health related quality of life
ILAR: International League of Associations for Rheumatology
IPAQ-SF: International Physical Activity Questionnaire, short form
IQR: Interquartile range
JAFS: Juvenile Arthritis Functionality Scale (JAMAR physical function domain)
JAMAR: Juvenile Arthritis Multidimensional Assessment Report
JDM: Juvenile dermatomyositis
JSLE: Juvenile systemic lupus erythematosus
MET: Metabolic equivalent of task
MVPA: Moderate to vigorous physical activity
PA: Physical activity
PGA: Physician Global Assessment (of disease activity)
PsA: Psoriatic arthritis
RPE: Rating of perceived exertion
SD: Standard deviation
SLE: Systemic lupus erythematosus
VO_2peak_: Peak oxygen uptake
