# Supplementary material for: Can fitness testing promote physical activity in adolescents with juvenile idiopathic arthritis?
Source: Pediatr Rheumatol Online J. 2026 May 21;24:46. doi: 10.1186/s12969-026-01223-6 (PMC13371447; doi:10.1186/s12969-026-01223-6)
Supplement: Supplementary file 1 — Supplementary Material 1 [file 12969_2026_1223_MOESM1_ESM.docx]

| Supplementary Table S1. Demographic characteristics | | | |
| --- | --- | --- | --- |
|  | Patients with complete dataset (n=24) | Patients with data at visit 1 only (n=10) | P-value |
| IPAQ |  |  |  |
| Walking, MET-minutes per day | 58 (44, 186) | 84 (5, 152) | 0.78 |
| Moderate MET-minutes per day | 69 (14, 137) | 69 (41, 103) | 0.75 |
| Vigorous PA, MET-minutes per day | 223 (59, 493) | 171 (94, 257) | 0.32 |
| MVPA MET-minutes, per day | 351 (166, 623) | 264 (180, 326) | 0.25 |
| Total PA MET-minutes, per day | 481 (192, 815) | 325 (229, 428) | 0.67 |
| Numbers are median (25^th^-75^th^ percentiles).  *IPAQ* The International Physical Activity Questionnaire, *MET* metabolic equivalent of task, *MVPA* moderate-to-vigorous physical activity, *PA* physical activity. | | | |

| Supplementary Table S2. Changes in MET-minutes per day | | | | |
| --- | --- | --- | --- | --- |
|  | Visit 1  (n=24) | Visit 2  (n=24) | Change | p-value |
| IPAQ |  |  |  |  |
| Walking, MET-minutes per day | 58 (44, 186) | 45 (21, 92) | -17 (-62, 49) | 0.520 |
| Moderate MET-minutes per day | 69 (14, 137) | 77 (11, 135) | -10 (-86, 71) | 0.543 |
| Vigorous PA, MET-minutes per day | 223 (59, 493) | 309 (103, 489) | 106 (-56, 290) | 0.476 |
| MVPA MET-minutes, per day | 351 (166, 623) | 446 (124, 607) | -40 (-177, 264) | 0.607 |
| Total PA MET-minutes, per day | 481 (192, 815) | 558 (169, 721) | 15 (-227, 246) | 0.909 |
| Numbers are median (25^th^-75^th^ percentiles).  *IPAQ* The International Physical Activity Questionnaire, *MET* metabolic equivalent of task, *MVPA* moderate-to-vigorous physical activity, *PA* physical activity. | | | | |

| Supplementary Table S3. Associations between MET-minutes, estimated VO_2peak_and walking distance | | | | |
| --- | --- | --- | --- | --- |
|  | Baseline (Visit 1) (n=34) | | Follow-up (Visit 2) (n=24) | |
|  | Estimated VO_2peak_  (mL∙kg^-1^∙min^-1^) | Walking distance (m) | Estimated VO_2peak_  (mL∙kg^-1^∙min^-1^) | Walking distance (m) |
| Walking, MET-minutes per day | 0.11 | 0.06 | 0.04 | 0.13 |
| Moderate MET-minutes per day | -0.10 | 0.14 | 0.08 | 0.08 |
| Vigorous PA, MET-minutes per day | **0.36*** | 0.17 | **0.51*** | **0.42*** |
| MVPA MET-minutes, per day | **0.32** | 0.23 | **0.50*** | **0.40** |
| Total PA MET-minutes, per day | **0.37*** | 0.27 | **0.45*** | **0.40** |
| Numbers are Spearman’s Rho, moderate correlations in bold, *p<0.05  *MVPA* moderate-to-vigorous physical activity, *PA* physical activity, *VO_2peak_* peak oxygen uptake | | | | |

| Supplementary Table S4. Associations between changes in MET-minutes, changes in estimated VO_2peak_ and changes in walking distance between visit 1 and visit 2 | | | | |
| --- | --- | --- | --- | --- |
| (n=24) | Estimated VO_2peak_  (mL∙kg^-1^∙min^-1^) | | Walking distance (m) | |
|  | Spearman’s rho | p-value | Spearman’s rho | p-value |
| Walking, MET-minutes per day | 0.24 | 0.27 | 0.15 | 0.48 |
| Moderate MET-minutes per day | -0.07 | 0.74 | 0.04 | 0.85 |
| Vigorous PA, MET-minutes per day | 0.06 | 0.77 | -0.01 | 0.96 |
| MVPA MET-minutes, per day | 0.05 | 0.81 | -0.02 | 0.92 |
| Total PA MET-minutes, per day | 0.14 | 0.52 | 0.04 | 0.84 |
| *MET* metabolic equivalent of task, *MVPA* moderate-to-vigorous physical activity, *PA* physical activity, *VO_2peak_* peak oxygen uptake | | | | |
